# Effects of Diets Containing Extruded Linseed and *Padina pavonica* Algae on Meat Rabbit: Carcass Performance and Meat Quality

**DOI:** 10.3390/foods14020274

**Published:** 2025-01-16

**Authors:** Nour Elhouda Fehri, Michela Contò, Marta Castrica, Alda Quattrone, Gianluca Renzi, Sabrina Di Giovanni, Stella Agradi, Daniele Vigo, Gabriele Brecchia, Laura Menchetti, Claudia Maria Balzaretti, Doriana Beqiraj, Egon Andoni, Giulio Curone, Sebastiana Failla

**Affiliations:** 1 Department of Veterinary Medicine and Animal Sciences, University of Milan, Via dell’Università 6, 26900 Lodi, Italy; nour.fehri@unimi.it (N.E.F.); alda.quattrone@unimi.it (A.Q.); daniele.vigo@unimi.it (D.V.); gabriele.brecchia@unimi.it (G.B.); claudia.balzaretti@unimi.it (C.M.B.); giulio.curone@unimi.it (G.C.); 2 Consiglio per la Ricerca in Agricoltura e l’Analisi Dell’Economia Agraria (CREA), Centro di Ricerca Zootecnia e Acquacoltura, Research Centre for Animal Production and Aquaculture, Via Salaria 31, 00015 Rome, Italy; michela.conto@crea.gov.it (M.C.); gianluca.renzi@crea.gov.it (G.R.); sabrina.digiovanni@crea.gov.it (S.D.G.); sebastiana.failla@crea.gov.it (S.F.); 3 Department of Comparative Biomedicine and Food Science, University of Padova, Agripolis, Viale dell’Univesità 16, 35020 Legnaro, Italy; 4 Department of Veterinary Sciences, University of Torino, Largo Paolo Braccini 2, 10095 Grugliasco, Italy; stella.agradi@unito.it; 5 School of Biosciences and Veterinary Medicine, University of Camerino, Via Circonvallazione 93/95, 62024 Matelica, Italy; laura.menchetti@unicam.it; 6 Faculty of Veterinary Medicine, Agricultural University of Tirana, Kodër Kamëz, 1029 Tirana, Albania; dkalamishi@yahoo.com (D.B.); eandoni@ubt.edu.al (E.A.)

**Keywords:** extruded linseed, *Padina pavonica*, meat, rabbit, PUFA, vitamin E

## Abstract

This study investigated the effects of dietary supplementation with extruded linseed (ELS) and *Padina pavonica* algae extract (PP) on rabbit carcass and meat quality. Ninety-six rabbit carcasses from two production cycles were analyzed. In the first cycle (C1), rabbits were fed a control diet (1CNT), the same diet supplemented with 5% ELS (1ELS5%), and supplemented with 3.5% ELS and 0.2% PP (1LPP3.5%). In the second cycle (C2), the diets varied in composition and supplementation levels: a different control diet (2CNT), the same diet with 5% ELS (2ELS5%), and with 5% ELS and 0.2% PP (2LPP5%). Meat analyses were performed on *Longissimus thoracis* et *lumborum* (LTL) muscle for physical properties and on thigh meat (THM) for proximate composition, vitamin E, coenzyme-Q10, cholesterol, fatty acid profile, and mineral content. No significant differences in LTL physical quality were observed in C1, although LTL was brighter in C2 (*p* < 0.001). THM in C2 had higher fat content (*p* < 0.001). Dietary supplementation with ELS and PP extract significantly increased polyunsaturated fatty acids (n-3 PUFAs) and improved the n-6/n-3 ratio (*p* < 0.001) in rabbit meat, demonstrating their positive impact on meat quality.

## 1. Introduction

Currently, the rabbit industry is currently facing a severe economic crisis, driven by structural weaknesses, a steady decline in consumer demand, and increasing criticism from Western consumers regarding animal welfare and ethical concerns [1]. Additionally, rabbit meat is priced higher than other meats, such as poultry or pork [2]. Despite these challenges, rabbit meat is a valuable source of bioactive compounds with potential nutraceutical benefits [3], offering exceptional nutritional advantages. It is rich in polyunsaturated fatty acids (PUFAs) [4], high-quality proteins, and essential amino acids [5], while providing moderate energy values, having low fat and cholesterol, and being an excellent source of B vitamins, particularly B12. Rabbit meat is also low in sodium [6] but high in phosphorus [3,7]. However, limited consumer awareness and inefficiencies in marketing and communication often overshadow these benefits. To enhance both consumer acceptance and economic viability, the rabbit meat industry must focus on innovative marketing strategies and better dissemination of its health benefits [6].

Moreover, to address and mitigate the health risks associated with unbalanced diets, research has increasingly focused on developing new functional foods by incorporating high-quality nutraceuticals into the animals’ diets [8]. This approach is particularly effective in monogastric species, such as poultry, rabbits, and pigs, where the absence of the rumen prevents microbial alterations of key nutrients, like vitamins and PUFAs. As a result, these nutrients can be directly incorporated into muscle tissue without significant modification [9,10,11,12]. Beyond enriching animal products, these dietary supplements also improve animal health by reducing oxidative stress and limiting the formation of free radicals [13]. These reactive compounds, if transferred to animal-derived foods, can negatively impact human health. This interconnected relationship highlights the “One Health” concept, which emphasizes the intrinsic link between the proper management of animal health, nutrition, and living conditions (key components of animal welfare) and human well-being [13].

Recent research has examined the effects of dietary PUFA supplementation on the growth performance and meat quality of rabbits [14]. Specifically, studies [15,16] have shown that dietary intake significantly influences the fatty acid composition of both fat depots and intramuscular fat. Furthermore, increasing emphasis has been placed on dietary strategies to enhance the n-3 PUFA content in rabbit feed, aiming not only to improve animal performance but also to meet the growing demand for these essential fatty acids in human diets. These efforts align with recommendations from the Food and Agriculture Organization (FAO) and the World Health Organization (WHO), which emphasize the importance of increasing n-3 intake while reducing n-6 consumption to achieve a balanced n-6/n-3 fatty acid ratio [17]. An imbalance in this ratio, combined with insufficient essential fatty acid intake in modern diets, has been associated with the rising prevalence of cardiovascular diseases in developed countries [17].

Rabbit diets have been enriched with various sources of n-3 polyunsaturated fatty acids (PUFAs) to enhance the nutritional value of their meat as a functional food. Among the strategies employed, linseed has been widely studied as a primary source of alpha-linolenic acid (ALA) [18]. Supplementing rabbit diets with 8% linseed significantly improved meat quality by increasing n-3 PUFA levels, and reducing the n-6/n-3 ratio. Other studies found that 3% linseed supplementation similarly enhanced the nutritional quality of rabbit meat without compromising product characteristics [19]. However, higher supplementation levels (6% or 9%) were associated with increased meat oxidation and cooking losses, although sensory acceptability remained unaffected after storage [20].

On the other hand, fewer studies have investigated the use of fish oil [21,22] and marine algae supplementation as sources of n-3 PUFAs in rabbit diets [23,24,25,26,27], with results varying significantly depending on the specific type of algae incorporated. Despite the long-established use of marine algae as a feed additive in livestock production [28], research specifically focusing on the application of *Padina pavonica* in animal diets remains limited [29].

In general, marine algae possess specific enzymatic pathways [30,31] that enable them to synthesize long-chain polyunsaturated fatty acids (PUFAs), including arachidonic acid (20:4 n-6), eicosapentaenoic acid (20:5 n-3), and docosahexaenoic acid (22:6 n-3). Among the advantages of incorporating seaweed into animal feed is its ability to exert significant biological effects, even at low inclusion levels, due to the presence of numerous bioactive antioxidant and antimicrobial compounds that support critical cellular functions [32]. Specifically, *Padina pavonica*, a brown alga (*Phaeophyta*), is known for its richness in bioactive compounds that enhance the absorption of phosphorus and calcium. Additional benefits are attributed to its variety of phenolic compounds and flavonoids, such as myricetin, quercetin, and resveratrol [33], along with fat-soluble vitamins like vitamins E and D, and water-soluble vitamins such as niacin, thiamine, pantothenic acid, and folic acid. Furthermore, *Padina pavonica* is abundant in phytosterols, including fucosterol, ergosterol, and β-sitosterol, which are vital for cellular functions and contribute to cholesterol reduction [32]. Its antifungal properties are also notable, as they can inhibit the growth of foodborne pathogens and spoilage microorganisms, including mycotoxin-producing species such as *Aspergillus*, *Fusarium*, and *Penicillium* [33]. This inhibition offers dual benefits: preventing food spoilage and reducing exposure to harmful mycotoxins, such as aflatoxins and ochratoxin A, which are among the most concerning toxins.

Supplementing rabbit diets with marine algae could provide numerous benefits for rabbits, acting as a detoxifying agent and promoting the accumulation of nutraceutical compounds in the meat, including antioxidants, fat-soluble vitamins, and essential minerals. Additionally, it helps reduce cholesterol accumulation and supports the production of high levels of very long-chain polyunsaturated fatty acids (VLC-PUFAs) of both the n-3 and n-6 series [30,32,33,34].

The present study aims to evaluate the effects of dietary supplementation with extruded linseed (ELS) and *Padina pavonica* algae (PP) extract on rabbit meat quality and carcass traits. Given their unique properties, these supplements are expected to influence carcass performance and nutritional quality of rabbit meat.

## 2. Materials and Methods

### 2.1. Animals and Diets

This study was conducted as part of the PRIMA project entitled “Omega Rabbit: food for health BenefIT”, funded by the European Union. The experimental trial received approval from the Italian Ministry and the Ethical Committee of the Department of Veterinary Medicine of the University of Milano (OPBA_18_2021). The experimental trial took place in a commercial rabbit farm located in Central Italy. The rabbits were reared in compliance with Legislative Decree No. 146, implementing Directive 98/58/EC, regarding the minimum standards for the protection of animals kept for farming purposes. Efforts were made to reduce animal distress and use only the minimum number of animals required to ensure reliable and consistent results. Additionally, the health and welfare of the rabbits were monitored daily by the farm’s responsible veterinarian.

The experiment was conducted over two separate rearing cycles, each carried out in a different year. For each cycle, the trial encompassed four weekly sessions, with 36 male New Zealand White rabbits included in each session, resulting in a total of 144 rabbits per cycle. After weaning (at 35 days of age), the animals had an initial weight of 816 g. The rabbits were housed in conventional individual cages (L × W × H: 75 × 38 × 25 cm) and were kept in a controlled environment, where temperature was maintained between +18 °C and +23 °C, relative humidity was kept between 60% and 75%, and the lighting cycle consisted of 16 h of light followed by 8 h of darkness. The animals were randomly divided in three experimental groups (n = 48 animals for each group per cycle), each receiving a distinct pelleted diet. The experimental diets were formulated based on current nutritional recommendations [35] for fattening rabbits, with the ingredient composition detailed in Table 1.

In the first cycle (C1), the three groups received three different diets formulated as follows: 1CNT = control diet; 1ELS5% = 1CNT supplemented with 5% ELS, and 1LPP3.5% = 1CNT supplemented with 3.5% ELS and 0.2% PP algae extract.

In the second cycle (C2), the CNT diet was modified in the fatty acid profile by increasing monounsaturated fatty acid (MUFA) levels and reducing n-6 PUFA content to improve fatty acid characteristics. In addition, the group that received PP was supplemented with 5% of ELS. Despite these modifications, the proximate composition and fiber content were consistent across all diets. Therefore, the groups of the second cycle received the diets formulated as follows: 2CNT = control diet; 2ELS5% = 2CNT supplemented with 5% ELS; 2LPP5% = 2CNT supplemented with 5% ELS and 0.2% PP extract. All the ingredients were adjusted to achieve isoenergetic and isoproteic formulations. The chemical composition and fatty acid profiles of the six diets are detailed in Table 2 and Table 3. Rabbits were slaughtered at 85 days of age. During the study (35 to 85 days of age), the rabbits were provided with a daily ration gradually increasing from 100 g/day to 160 g/day. Fresh water was always available.

### 2.2. Carcass Dissection and Meat Sampling

Twelve rabbits from each group were individually weighed before slaughter during each weekly session. The handling of animals during the trial adhered to Legislative Decree No. 146, which enforces Directive 98/58/EC. The slaughtering process was conducted in a certified slaughterhouse, located on the same farm, so there was no transport involved for the animals. The animals were stunned by electronarcosis. Carcasses were immediately chilled at 4 °C, and four carcasses per group were randomly selected for analysis, resulting in a total of 64 carcasses per cycle (16 per experimental group). Each week, the 12 selected carcasses (without head) were transported in refrigerated boxes to CREA-ZA laboratory. Twenty-four hours after slaughter, the carcasses were weighed to determine refrigerated carcass weight and were dissected according to the recommendations of the Worlds Rabbit Science Association (WRSA) [36]. The weights of the carcass excluding the liver, lungs, thymus, esophagus, heart, and kidneys were recorded as the reference carcass weight (RCW). Additionally, the weights of the liver (LvW), kidneys (KiW), abdominal fat (AFW), and scapular fat (SFW), as well as the percentage of the carcass regions defined as fore leg (FLP), thoracic part (TP), and hind part (HPP), were also recorded. For each carcass, the two *Longissimus thoracis et lumborum* (LTL) muscles were collected for physical analyses, including pH, color, drip and cooking loss, and shear force on cooked meat. The right hind leg was carefully dissected to separate and weigh the bone, muscle, and the combined intermuscular fat and connective tissues. This process allowed for the estimation of the percentage distribution of tissues, classified as hind leg bone percentage (HLBP), hind leg meat percentage (HLMP), and hind leg fat and other tissue percentage (HLFP). Additionally, the meat-to-bone ratio (M/B) of the hind leg was calculated using the formula: M/B = HLMP/HLBP. A sample from the thigh was obtained during dissection from the first portion of muscles near the distal part of the femur, as this area contains several muscles composed of both white and red fibers. The hind leg muscles, after removing the visible fat and tendons (THM), were finely ground under controlled conditions at 4 °C to prevent overheating.

Fresh thigh samples were analyzed for proximate composition, including moisture, protein, fat, and ash content (expressed as g/100 g of meat). The remaining samples were vacuum-packed and frozen at −80 °C for subsequent chemical analyses, including vitamin E, coenzyme Q10, cholesterol content, fatty acids profile, and mineral concentrations (macroelements: phosphorus, potassium, sodium, magnesium; microelements: iron, copper, zinc).

### 2.3. Physical Analysis

#### 2.3.1. pH

Immediately after dissection, two samples of 1 g of minced LTL muscle were mixed with 10 mL of NaCl solution (0.1 mol/L) at pH 7. The mixture was homogenized using a homogenizer (T 25 digital ULTRA-TURRAX^®^—IKA) for 30 s at 6000 rpm. The pH of each sample was measured using a digital pH meter with temperature compensation (XS Instrument Serie 80 PC80, Giorgio Bormac S.r.l., Carpi, Italy), calibrated at pH 7 and pH 4. The final value was obtained as the mean of the two measurements.

#### 2.3.2. Color

Color was recorded on the LTL muscle using the CIELAB system [37] to estimate lightness (L*), redness (a*), and yellowness (b*), with D illuminant (6504°K, daylight) using a Konica Minolta CM-3600 D (Sensing, Inc., Osaka, Japan) color spectrophotometer. The muscle was cut along its longitudinal line and maintained to air for 30 min to allow a blooming effect. Color data were obtained as the means of four measurements per sample.

#### 2.3.3. Water Holding Capacity

Water holding capacity (WHC) in the LTL muscle was determined using two samples of approximately 1 g each. The samples were finely minced, placed between two paper discs, and subjected to an external force of 1 kg for 5 min. Following compression, the samples were reweighed, and the weight difference was expressed as a percentage of WHC. This analysis was performed in duplicate [38].

Cooking loss was evaluated according to the method described by Honikel and Hamm [38]. Two weighed portions of about 5 cm, one from each LTL muscle (right and left) at the same anatomical position, were sealed in plastic bags under light vacuum conditions and cooked in a water bath until reaching 75 °C at the core of the sample for approximately 20 min and then cooked in ice water. From this determination, we use a portion of both LTL muscles measuring approximately 5 cm in length.

#### 2.3.4. Shear Force

Shear force was determined using a Warner–Bratzler Shear (WBS) apparatus on a dynamometer (Instron 5543 Single Column Table Top Tensile Tester System, Instron, Norwood, MA, USA) on cooked meat samples measuring 1 × 1 cm in thickness and 2 cm in length, cut along the fiber direction of muscles. The speed of the crosshead was set at 100 mm/min, as reported in Contò et al. [39]. Cooked meat samples were obtained as described above for the cooking losses on LTL muscles. The final shear force value was the mean of four determinations and expressed in Newton (N).

### 2.4. Chemical Analysis

#### 2.4.1. Proximate Composition

The proximate composition of THM and pellet was analyzed according to AOAC [40] methods for dry matter (DM, method 934.01), ether extract (EE, method 920.39), ash (method 942.05) and crude protein (CP, method 984.13). Immediately after dissection, 8 g of samples was used for dry matter determination at 102 °C for 24 h. On the same sample, the ash % was determined at 540 °C for 8 h. Ether extract was identified using a traditional Soxhlet extraction method with diethyl ether (Tecator Soxtec System HT 1043 extraction unit Gemini, Apeldoorn, Sweden). Protein content (N × 6.25) was measured using the Kjeldahl method with Tecator Digestion System and Kjeltec Auto 1030 Analyzer (FOSS, Hillerød, Denmark).

#### 2.4.2. Vitamin E

Vitamin E (tocopherol) was extracted from minced THM after saponification phase with 60% KOH at 70 °C per 1 h, as reported in Gąsior et al. [41]. The vitamin was recovered with hexane/ethyl acetate (9:1 vol:vol), dried and suspended in methanol. The extract was injected into an HPLC Alliance 2695, Waters (Waters Corporation, Framingham, MA, USA) with a Kinetex 5 µm EVO (Phenomenex, Torrance, CA, USA) C18 column with isocratic mobile phase, methanol/Waters (95:5 vol:vol) and read in fluorescence at λEX = 295 nm, λEM = 350 nm. The vitamin E peak was identified by comparison with the standard tocopherol peak and expressed as µg/g of meat [42].

#### 2.4.3. Coenzyme Q10

The analysis of coenzyme Q10 was performed on minced THM following the method described by Mattila et al. [43]. Briefly, 1 g of meat was homogenized in a sodium chloride solution, and the sample was washed twice with an organic solvent mixture (ethanol/n-hexane 1:1 *v*/*v*) to separate the coenzyme Q10. The extract was then filtered, evaporated, and re-suspended in n-propanol. A sample of 20 µL was recovered and injected into an HPLC Alliance 2695, Waters (Waters Corporation, Framingham, MA, USA), with a Gemini NX 5 µm (Phenomenex, Torrance, CA, USA) C18 column. The isocratic phase consisted (methanol/ethanol/2-propanol/ammonium acetate buffer at concentrations of 53:21:21:1, respectively), and detection was performed at 275 nm. The peak representing the coenzyme Q10 was identified compared to the coenzyme Q10 standard peak and expressed as mg of Q10/100 g of meat.

#### 2.4.4. Cholesterol

Cholesterol content was performed following the procedures of Maraschiello et al. [44]. Briefly, 0.1 g of minced THMt was saponified with 0.5 N KOH methanolic solution, with BHT, at 80 °C for 1 h. After cooling, a saturated sodium chloride solution was added, and the unsaponifiable fraction, containing cholesterol, was extracted twice with a mixture of ethylic ether/hexane (1:1 vol:vol). Subsequently, the extracted fraction was then dried under nitrogen flow and re-suspended in acetonitrile/isopropanol (1:1 vol:vol) and injected in HPLC Alliance 2695, Waters (Waters Corporation, Framingham, MA, USA) with Synergi 4µ (Phenomenex, Torrance, CA, USA) C18 column with isocratic phase acetonitrile/isopropanol (55:45 vol:vol) and read at 210 nm. The cholesterol was identified by comparing the sample peak with standard cholesterol peak and expressed in mg/100 g of meat.

#### 2.4.5. Fatty Acids Profile

The fat was extracted from approximately 6 g of minced THM or 2 g of pellet using a chloroform/methanol mixture (2:1 vol:vol). After suspension in hexane, the fat was methylated following the IUPAC procedure [45] using a 2 M of methanolic KOH solution, obtaining fatty acid methyl esters (FAMEs) solubilized in hexane. The FAMEs were quantified using a gas-chromatography (GC) system (GC 6890N, Agilent, Inc., Santa Clara, CA, USA) equipped with a flame ionization detector (FID) and a CP-Sil88 fused silica capillary column (100 m 0.25 mm (internal diameter) with 0.2 μm film thickness; Agilent Technologies). Fatty acid extraction, methylation, and gas chromatograph conditions were reported in detail by Failla et al. [46]. An internal standard C19:0 was added to the samples before the fatty acid extraction.

Fatty acid methyl esters were identified by comparing the retention time peaks of each compound with standard peaks from the Supelco mix 37 FAME and docosapentaenoic acid (Sigma-Aldrich Merck, Darmstadt, Germany). The different classes of fatty acids, including saturated fatty acids (SFAs), MUFAs, PUFAs, PUFA n-6, PUFA n-3 and n-6/n-3 ratio, were calculated and expressed as a % of total FAMEs.

#### 2.4.6. Mineral Content

The mineral composition was estimated using Near-Infrared Spectroscopy (NIR). Samples of THM were lyophilized, and the NIR spectra were acquired using the NIRFLEX 500 (Büchi, Flawil, Switzerland). The data were processed using a previously developed Partial Least Squares (PLS) model, which was calibrated on 120 rabbit meat samples. Before application, the model was revalidated using 18 meat samples (three from each diet group), for which mineral content was analyzed following the methods specified below.

Minerals such as potassium, sodium, iron, copper and zinc were analyzed using PerkinElmer atomic absorption spectroscopy (AAS, PerkinElmer, Shelton, CT, USA) and a microwave Ethos 900 (Milestone, Shelton, CT, USA) for the mineralizing phase. Samples of 1 g of meat were mineralized with 64% nitric acid and hydrogen peroxide using the Ethos 900 microwave, and the solution was then read in AAS using a multi-channel lamp. A characteristic wavelength for each mineral was used to measure absorbance, and the results were expressed as mg/100 g or mg/kg of meat for microelements. For phosphorus, a photometric procedure was used [47]. After mineralization phase, the sample was reacted with a molybdate reagent (1%) and hydrazine sulfate (0.2%), and the phosphorus content was read and quantified in a PerkinElmer spectrophotometer Lambda 25 (PerkinElmer, Shelton, CT, USA) at 730 nm, with results expressed as mg/100 g of meat.

The dataset obtained from these analyses was used as a validation set. The results showed a coefficient of determination (R^2^) greater than 0.83 for all minerals, with an RPD (Ratio of Performance to Deviation) of approximately 3. The RPD was calculated as SD/SEP × 100, where SD is the standard deviation of the mineral data and SEP is the standard error of prediction.

### 2.5. Additional Chemical Analysis on Pelleted Feed

A sample of about 1 kg was finely ground using a mill (Cemotec 1090 sample mill, FOSS, Hilleroed, DK, EU), to obtain a uniform sample both in composition and particle size. The following analytical determinations were performed on feedstuff used as proximate composition, including dry matter (DM), ash, crude protein (CP), and crude fat (EE), using the same methods and instruments as those described for meat analysis [48]. Calcium, phosphorus, vitamin E, and fatty acids were also analyzed following the procedures described for meat samples [41].

#### 2.5.1. Crude Fiber

Crude fiber (CF) was determined as reported in AOAC [40] methods 962.09 ISO 5498:2000, using a fiber analyzer (FIWE Fiber Extractor, Velp Scientifica, New York, NY, USA). A minced feed sample of 1 g ± 0.1 was weighed and placed in a pre-weighed crucible and then boiled in 0.12 M sulfuric acid for 30 min. Afterward, the sample was washed with distilled water to neutralize the pH and remove residues. The sample was then digested in a 0.22 M alkaline f potassium hydroxide solution. After alkaline digestion, the sample was washed with distilled water to neutralize the pH, and then defatted with acetone. The crucible was dried in an oven at 100 °C, and the weight was recorded. The sample was incinerated in an oven at 550 °C for 2 h and weighed again. CF is expressed in g/100 g of sample.

#### 2.5.2. Starch

Starch was quantified by the enzymatic colorimetric method as reported in AOAC method 996.11 [40]. The procedure was performed using an assay kit from Megazyme (Megazyme International, Wicklow, IE, EU). Briefly, 100 mg of feed was mixed with an 80% ethanol solution (vol:vol), followed by the addition of 3 mL of α-amylase and incubation for 11 min. Afterward, 0.1 mL of amyloglucosidase was added, and the sample was incubated again. The sample was then centrifuged, and a portion of the supernatant was incubated for 20 min at 50 °C. The starch content was read at 510 nm, and expressed as g/100 g of sample.

#### 2.5.3. Amino Acids

Total amino acids were determined using AccQ•Tag Fluor reagent kit (Waters, Milford, MA, USA) [Water manual] on an HPLC system (Waters Alliance 2695, Waters Corporation, Framingham, MA, USA) with an AccQ•Tag column (3.9 × 150 mm, Waters Corporation, Framingham, MA, USA). A sample of about 1 g was hydrolysed with HCl 6 N for 14 h at 110 °C, except for tryptophan, for which NaOH 4 M was used. After hydrolysis, the samples were appropriately diluted and derivatized with the AccQ•Tag reagent, then detected by fluorescence at λEX = 205 nm and λEM = 395 nm. The gradient mobile phase consisted of Waters buffer/acetonitrile/water (vol:vol:vol). Peak identification was performed by comparing the sample peaks with the standard peaks from the AccQ•Tag kit, and results were expressed as g/100 g of meat.

#### 2.5.4. Nitrogen-Free Extract and Digestible Energy

The nitrogen-free extract (NFE) content was calculated according to the following formula:

NFE(g/100) = DM − (CF + EE + CF + ash)(1)

Based on the identified chemical composition, the digestible energy (kcal/kg) of the feed was calculated using the equation provided by the EU guidelines for rabbit feed [48].

Digestible energy (kcal/kg) = −1801 + 7.10 CP + 12.01 × EE + 5.59 × NFE(2)

### 2.6. Statistical Analysis

To evaluate the differences among the six groups of rabbits, a mixed model was employed, incorporating two fixed factors and their interaction, with all physical and chemical composition data used as dependent variables. In this model, feed group and cycle were treated as fixed factors, while the weekly session was considered a random effect. The analysis was conducted using the MIXED procedure, on the SAS statistical package (SAS Institute Inc., Cary, NC, USA). Mean comparisons were performed using the Student’s *t*-test, with the significance threshold set at *p* < 0.05. This approach ensures a robust and statistically sound evaluation of the physical and chemical composition differences across feed groups while accounting for potential confounding factors and individual variability.

## 3. Results and Discussion

### 3.1. Carcass Characteristics

The data presented in Table 4 provide a comparative analysis of live weight and carcass characteristics of rabbits fed different diets across two cycles. During the growth stage, the rabbits had an average daily weight gain of 32.4 ± 3.2 g for C1 and 35.6 ± 2.8 g for C2 across the three experimental groups. Daily pellet consumption averaged 133.5 g, with no significant differences observed between cycles or groups.

Rabbit live weight (LW) in C1 ranged from 2398 g (1ELS5%) to 2477 g (1CNT), while in C2, it varied from 2467 g (2ELS5%) to 2541 g (2CNT). However, these differences were not statistically significant (*p* > 0.05) either among groups or between the two cycles. These results are consistent with those of Pla et al. [35], who did not observe detrimental effects of sunflower oil and linseed on rabbit LW. Similarly, the chilled carcass weight (CCW) in C1 ranged from 1345.3 g (1ELS5%) to 1390.1 g (1CNT), and in C2 from 1399.9 g (2ELS5%) to 1441.0 g (2CNT), with no significant differences observed among the groups and cycles. However, slight variability was observed between cycles for CCW, in which C2 reported a trend (*p* = 0.086) higher than C1 (1425.8 g vs. 1368.4 in average for the groups, respectively, for C2 vs. C1).

The LvW, SFaW, and PFaW did not show significant differences among groups in either cycle. The average values for all groups were 79.6 ± 11.7 g for liver weight, 15.3 ± 1.5 g for scapular fat, and 7.9 ± 2.6 g for perirenal fat. Regarding carcass composition, significant differences were observed between cycles for fore leg (FLP) and hind part (HPP). However, in C2, the groups receiving ELS supplementation (2ELS5% and 2LPP5%) tended to show a higher percentage of HPP (*p* = 0.063).

The tissue composition of the thighs showed significant differences in HLFP between cycles, with all three groups in C2 exhibiting higher values (*p* = 0.042). For HLBP and HLMP, significant differences were observed among groups only in C2 (*p* = 0.002 and *p* = 0.015, respectively). The 2CNT group exhibited lower HLBP and higher HLMP compared to the other groups. This trend also impacted the M/B ratio, where significant differences were observed in C2, with the 2CNT group showing a significantly higher M/B ratio (*p* = 0.005) compared to the other two groups. This observed tendency toward greater muscle trophism in the control group in C2, reflected in the enhanced development of the HPP region, was due to the higher dietary intake of oleic and linoleic acids, which appear to have a confirmed ability to promote muscle growth as reviewed by Abreu et al. [49]

In contrast, Dal Bosco et al. [50] reported that whole linseed supplementation had no effect on rabbit carcass traits and that the diet did not influence the meat-to-bone ratio (4.06 ± 0.06) or the percentage of perirenal (3.33 ± 0.16%) and scapular fat (0.76 ± 0.03%).

In this study, the supplemented diets had a limited impact on overall rabbit growth but contributed to the enhancement of specific high-value carcass traits, such as muscle development, particularly in the C2. This effect is likely attributable to the lower linoleic acid content and the higher oleic fatty acid levels in the C2 diets. These findings highlight the importance of long-term dietary strategies and targeted nutritional interventions in enhancing rabbit meat production. Moreover, these results align with previous studies on the inclusion of n-3 PUFA-rich vegetable sources in rabbit diets, which have consistently shown no significant impact on major carcass traits [35,50].

Further investigation is needed to better understand the relationship between diet and meat quality, particularly concerning the bioavailability of nutrients from experimental feed ingredients [1].

### 3.2. Physical Characteristics of LTL of Rabbit Feed on Different Diets

Table 5 shows the physical characteristics of the LTL muscle from rabbits fed with different diets across two rearing cycles. In both cycles, the different diets did not significantly affect the physical composition of this muscle. These data agreed with some authors that reported no linseed effect on rabbit meat physical analyses [20]. However, when comparing the two cycles, the groups of C2 on average showed higher values for pH, L* (lightness), and WHC (*p*= 0.002, 0.001, and 0.002, respectively). In contrast, lower values were observed for cooking loss and shear force in cooked meat (*p* = 0.014 and *p* = 0.018, respectively).

The pH of meat plays a crucial role in influencing various quality attributes, such as WHC, color, and shear force [51]. In this study, pH values remained consistent across groups within each cycle, indicating that the diets containing ELS and PP extract did not affect the muscle’s post-mortem biochemical properties. These pH values were similar to those reported by Mattioli et al. [52] on rabbits fed diets supplemented with linseed. In our study, the significant difference in pH between cycles suggests that pre- or post-slaughter factors may have influenced this parameter [51].

The WHC was lower in C1 than in C2 (13.28% vs. 14.20%, respectively, as the average of the three groups). This difference was likely due to the higher pH of C2 meat, which may have affected protein structure and degradation, consequently influencing WHC [4]. As a result, cooking loss was lower in C2 than in C1 (27.49% vs. 26.59%, on average for the groups in each cycle), likely due to the higher drip loss in C2.

In general, meat pH and lightness are negatively correlated. However, in the LTL of the C2, despite having a significantly higher pH, greater lightness was observed. The reason for this may lie in the changes induced by the diet. Specifically, the higher percentage of MUFAs and the lower levels of n-6 PUFAs in the diets of the second cycle increased the fat content, which is known to result in a paler meat color [53]. Color is usually an indicator of freshness and safety, influencing consumer purchase intention [54,55]. The higher presence of fat in the animals and a higher pH that promote greater proteolysis likely affected the WBS values of cooked meat (*p* = 0.014), making the LTM more tender in the second cycle compared to the first.

Previous studies on rabbit meat quality following dietary supplementation with linseed [56] and brown algae [57] found no significant effects of these ingredients on meat color [18,53,58].

### 3.3. Chemical Characteristics

The chemical composition of thigh meat is reported in Table 6. In both C1 and C2, no significant differences were observed in proximate composition among groups.

Between the two cycles, the percentage of ash and fat were significantly higher in C2 compared to C1 (*p* = 0.019 and 0.001, respectively), whereas moisture content was higher in C1 than C2 (75.57 vs. 74.98% in means for the three groups for each cycle). This trend was due to a negative correlation between moisture and fat percentage content [59]. These differences between the two cycles are consistent with the greater amount of HLFP observed in the thigh in C2, probably due to the higher percentage of MUFAs in the diet.

Significant differences were reported between diets for vitamin E and Q10 (Table 6), while for cholesterol, only a trend toward significance was observed (*p* = 0.109). Among the different diets within the same cycle, the highest vitamin E values were observed in the carcasses belonging to the rabbits fed with *PP* extract (*p* < 0.001 in both cycles). As reported by Caf et al. [33], marine algae such as *PP* are naturally rich in phenolic compounds, antioxidants, and fat-soluble vitamins, including vitamin E. Prates [60] found that algae significantly contribute to vitamin fortification, particularly vitamin E, which acts as an antioxidant to improve meat stability and shelf life. In this regard, Ribeiro et al. [61] reported that supplementing broiler diets with 2% microalgae increased vitamin E content in chicken meat, enhancing its nutritional value and health benefits for consumers.

In C1, coenzyme Q10 levels were higher in 1ELS5% compared to 1CNT (20.33 mg/100 g vs. 19.24 mg/100 g, *p* = 0.043), but not significantly different from 1LPP3.5%. In C2, both 2ELS5% and 2LPP5% showed the highest coenzyme Q10 levels compared to the 2CNT diet (20.44 mg/100 g as average of diets with ELS vs. 19.61 mg/100 g, *p* = 0.031). Previous studies have reported high concentrations of coenzyme Q10 in vegetables and algae. However, the inclusion of ELS and PP in the diets could explain the lower levels observed in the CNT diets [62].

In C1, cholesterol levels did not significantly differ among groups. However, in C2, the 2LPP5% diet tended to reduce cholesterol levels in the meat. The observed differences in cholesterol content in the 2LPP5% group may be due to the PP supplementation, although this effect may have been limited due to the low inclusion level of PP in the diet [32].

Similarly, Vlaicu et al. [63], in their study on the nutritional composition and bioactive compounds of basil, thyme, and sage, demonstrated that plant additives significantly reduced cholesterol concentration in broiler thigh meat. Martin et al. [64], in their study on the effect of dietary inclusion of Spirulina on meat quality traits in piglets, reported a reduction in cholesterol concentration.

These results reinforce the notion that incorporating bioactive-rich plant additives, such as PP and other functional compounds, into animal diets can improve meat nutritional quality by lowering cholesterol levels, thus meeting consumer expectations for healthier meat products.

### 3.4. Fatty Acid Composition

A fatty acid profile on THM revealed significant effects of diet and cycle on the principal fatty acid classes (Table 7). SFAs were the predominant components, although rabbit meat contains high amounts of MUFAs and PUFAs, compared to other livestock meats [65].

SFA levels were significantly lower in both cycles for rabbits fed the supplemented diets compared to their respective control groups. The lowest SFA levels were observed in the groups fed 2LPP5% (*p* < 0.001) followed by 2ELS5%, 1ELS5% and 1LPP3.5%.

In the C1, the THM muscle exhibited the highest SFA content among the three groups compared to C2 (*p* = 0.007) likely due to differences in the fatty acid composition of the diets between the two cycles. This change naturally led to a greater accumulation of MUFAs in rabbit meat of C2 compared to C1 (28.36% vs. 24.24%, respectively, on average of the three groups within cycles; *p* < 0.001). Significant differences in MUFA content among groups within each cycle were observed only in C2. The highest MUFA value was recorded for the 2CNT group, followed by progressively lower values for the 2ELS5% and 2LPP5% diets, ranging from 29.88% to 27.08%.

Contrasting percentages of n-6 PUFA were observed between the two cycles, with C1 showing a higher average across diets compared to C2 (32.52% vs. 27.04%, respectively, as average of the three groups within cycles). This difference was attributed to variations in the n-6 fatty acid composition of the diets used in the two cycles. Additionally, the n-6 PUFA percentage in THM was significantly influenced by groups within each cycle (*p* < 0.001), with the highest levels observed in the CNT groups of both cycles, followed by the PP-supplemented groups. Furthermore, differences in n-3 PUFA content were observed between cycles, although the increase was relatively modest (approximately +1 percentage point).

Similar trends have been reported in broiler meat, as shown by Ivanova et al. [66], where the inclusion of flaxseed oil in the diet decreased SFA levels and increased PUFA levels, particularly due to the higher content of linoleic and linolenic acids in the lipids. In their study, a diet supplemented with 3% flaxseed oil had the greatest influence on the lipid composition of broiler meat, aligning with the findings of our study.

The n-3 PUFA content in rabbit meat significantly increased (*p* < 0.001) in rabbits fed diets containing ELS and PP extract compared to CNT diets. These findings highlight the potential of linseed and algae supplementation to improve the lipid profile of rabbit meat, making it more beneficial for human health. The results are consistent with previous research showing that dietary n-3 PUFA supplementation can improve the fatty acid profile of rabbit meat [18,28,32,58,67]. For example, linseed supplementation in rabbits has been shown to significantly increase n-3 PUFA content while reducing SFA and MUFA levels in both the *longissimus dorsi* muscle and perirenal fat [68].

The total PUFA content showed lower differences in the C1 cycle, as the opposing trends observed for n-6 and n-3 PUFAs balanced each other out. In the C2 cycle, the large differences in n-3 PUFA content among groups influenced the total PUFA level, reflecting the trend of n-3 PUFA. The lowest total PUFA value was recorded in 2CNT, followed by 2ELS5%, with the highest value observed in 2LPP5%. Similarly, Marino et al. [69] observed that including linseed in the diet increased the content of n-3 PUFA and MUFA, while reducing the proportions of SFA and n-6 PUFA in beef meat. Likewise, Atti et al. [70] observed a significant increase in n-3 PUFA content in lamb meat with the incorporation of extruded linseed in the diet. These findings further support the efficacy of linseed supplementation in improving the lipid profile of meat across different species.

In this study, the n-6/n-3 ratio was significantly different between cycles and groups (*p* < 0.001 for both). This ratio is a crucial health indicator, and its reduction is considered beneficial for human health [50,71]. Notably, in rabbits fed diets supplemented with ELS, this ratio decreased from 9.05 in the 1CNT group to 2.68 as average of the other two groups. Although the 1ELS5% and 1LPP3.5% diets differed in ELS supplementation (5 vs. 3.5%), they did not show significant differences for this ratio. Similar reductions were observed in the second cycle (from 8.10 in the 2CNT to 2.02 on average for the other two groups).

These findings highlight the potential of linseed-enriched diets to improve the fatty acid profile of rabbit meat, particularly by increasing the relative proportion of n-3 PUFA, while lowering the n-6/n-3 ratio. A few several studies have investigated the impact of fish oil-enriched diets on the fatty acid composition of rabbit meat, consistently reporting a reduction in the n-6/n-3 ratio, with some values reaching as low as 1.61 [21,72,73]. These findings highlight the potential of dietary strategies aimed at reducing the n-6/n-3 ratio to improve the nutritional quality of rabbit meat for human consumption. In contrast, a study found a reduction in the n-6/n-3 ratio in lambs, which decreased from 5.23 in the control group to 0.71 and 1.1 in the group fed extruded linseed [70].

### 3.5. Macro- and Micromineral Elements

The analysis of macro- and micromineral content (Table 8) in rabbit THM revealed no significant differences in mineral content among groups, except for phosphorus, zinc, and copper between the two cycles. These results were anticipated, as the control diet was formulated to meet the recommended concentrations of macrominerals.

Phosphorus levels were significantly higher in the 2LPP5% diet compared to the control diet (246.92 mg/100 g vs. 238.92 mg/100 g) in C2. Zinc content also increased significantly with dietary supplementation in both cycles, with the highest levels observed in the 1LPP3.5% and 2LPP5% groups compared to their respective controls (12.26 mg/kg and 12.52 mg/kg vs. 11.27 mg/kg and 11.42 mg/kg, respectively). Marine macroalgae are well known for their high mineral content, particularly zinc [74]. Moreover, brown algae may have higher bioavailability of phosphorus, likely due to lower levels of anti-nutritional compounds, such as phytic acid [74]. Improved mineral bioavailability enhances the absorption and utilization of these nutrients by rabbits, potentially reducing the need for additional mineral supplementation in their diets. This approach could help maintain or even improve the nutritional quality of the meat [24].

Elevated levels of phosphorus and zinc not only enhance the nutritional value of rabbit meat but also align with the growing demand for functional foods that provide additional health benefits beyond basic nutrition. Furthermore, rabbit meat is uniquely characterized by its low sodium content and high levels of potassium and phosphorus compared to other meats such as pork, beef, and chicken [3,75]. These attributes make rabbit meat an excellent choice for diets aimed at preventing hypertension, further solidifying its role as a functional and health-promoting food [3].

## 4. Conclusions

This study demonstrates the significant effects of dietary supplementation with extruded linseed (ELS) and Padina pavonica (PP) algae extract on carcass performance and meat quality of rabbits. These dietary interventions notably improved the fatty acid profile of rabbit meat by reducing saturated fatty acids and the n-6/n-3 ratio, while increasing n-3 PUFA levels. Additionally, the supplemented diets enriched the meat with essential minerals, particularly phosphorus and zinc, and increased the levels of vitamin E and coenzyme Q10, enhancing both meat quality and its nutritional value. Meat analyses performed on *Longissimus thoracis et lumborum* (LTL) muscle and thigh meat (THM) revealed notable findings. While no significant differences in LTL physical quality were observed in the first production cycle, LTL brightness was significantly higher in the second cycle. Thigh meat in the second cycle also exhibited higher fat content. Importantly, the supplementation with ELS and PP extract demonstrated a consistent positive impact on the polyunsaturated fatty acid (PUFA) profile and the overall nutritional value of the meat. Although some variability was observed between the two breeding cycles, the results highlight the potential of these dietary strategies to produce healthier, nutrient-rich rabbit meat, offering significant benefits for human health.

## Figures and Tables

**Table 1:** Formulation of the experimental diets of the different groups in the two cycles.

Ingredients (%)	First Cycle			Second Cycle		
	1CNT	1ELS5%	1LPP3.5%	2CNT	2ELS5%	2LPP5%
Extruded linseed	-	5	3.5	-	5	5
“*Padina pavonica*” Algae extract	-	-	0.2	-	-	0.2
Wheat Bran	23.16	23.09	23.08	24	23	23
Beet pulp	11.5	9.33	11	13.5	12.5	11.48
Wheat straw	11	11	11	11	11	11
Alfalfa	10	12.5	10.17	10	10	10
Sunflower husks	9.95	6	10.78	-	-	-
Barley	9.5	9	9.5	5.6	5.8	8.25
Sunflower seed meal	8.83	14	9.17	18.5	17.41	17.97
Soybean hulls	0.17	-	-	3.82	4.3	2.22
Toasted soybean seed	5	-	1.50	1.7	-	-
Molasses cane	3	3	3	2.5	2.5	2.5
Wheat	2.5	2.5	2.5	3	3	3
Grape seed meal	2.17	1.83	1.87	2.7	2.8	2.68
Soybean oil	0.55	-	-	0.99	-	-
Palm oil	0.33	0.33	0.33	0.33	0.33	0.33
Carboxymethylcellulose	0.2	0.2	0.2	0.2	0.2	0.2
Liquid acidifier ^1^	0.15	0.15	0.15	0.15	0.15	0.15
Calcium carbonate	0.8	0.8	0.8	0.8	0.8	0.8
Sodium chloride	0.4	0.4	0.4	0.42	0.42	0.42
Magnesium oxide	0.15	0.15	0.15	0.15	0.15	0.15
Oligo-vitamin supplement ^2^	0.25	0.25	0.25	0.25	0.25	0.25
Methionine hydroxyanalog	0.15	0.14	0.15	0.15	0.13	0.12
Lysine	0.14	0.21	0.19	0.14	0.16	0.17
L Threonine	0.07	0.08	0.08	0.07	0.07	0.07
Vitamin E 50%	0.03	0.03	0.03	0.03	0.03	0.03

^1^ Liquid acidifier composition = Formic acid 75%. ^2^ Oligo-vitamin supplement: Vitamin A 6,000,000 IU, D3 600,000 IU, E 20,000 IU, K3 1200 mg, B1 800 mg, B2 1600 mg, B6 800 mg, B12 6.0 mg, biotin 60.0 mg, niacinamide 16,000 mg, folic acid 400 mg, calcium pantothenate 6666 mg.; 1CNT = control diet of the first cycle; 1ELS5% = 1CNT+ 5% ELS; 1LPP3.5% = 1CNT+ 3.5% ELS and 0.2% *PP* extract. 2CNT = control diet of second cycle; 2ELS5% = 2CNT+ 5% ELS; 2LPP5% = 2ELS5% with 5% ELS and 0.2% *PP* extract.

**Table 2:** Chemical composition of the experimental diets of the different groups in the two cycles.

Components g/100 g	First Cycle			Second Cycle		
	1CNT	1ELS5%	1LPP3.5%	2CNT	2ELS5%	2LPP5%
Dry matter	88.61	88.89	88.85	89.96	89.05	88.89
Crude proteins	15.03	15.28	15.13	15.02	15.25	15.11
Crude fat	3.56	3.75	3.57	3.74	3.81	3.88
Ash	7.95	8.04	8.10	7.88	7.95	8.09
Crude fiber	18.64	18.63	18.71	18.70	18.45	18.34
Nitrogen-free extract	43.43	43.19	432.34	43.62	43.59	43.47
Calcium	0.79	0.80	0.81	0.80	0.79	0.81
Phosphate	0.51	0.52	0.53	0.50	0.51	0.53
Starch	12.07	12.07	11.87	12.10	12.05	12.03
Digestible energy (kcal)	2189.1	2190.4	2188.7	2196.4	2198.7	2197.9
Met + Cys ^1^	0.6	0.6	0.6	0.6	0.6	0.6
Lys ^2^	0.7	0.7	0.7	0.7	0.7	0.7
Trp ^3^	0.19	0.19	0.19	0.19	0.19	0.19
Thr ^4^	0.58	0.58	0.58	0.58	0.58	0.58
Vitamin E (ppm)	200	200	200	200	200	200

^1^ Met + Cys = Methionine + cysteine; ^2^ Lys = lysine; ^3^ Trp = tryptophan; ^4^ Thr = threonine., 1CNT = control diet of first cycle; 1ELS5% = 1CNT+ 5% ELS; 1LPP3.5% = 1CNT+ 3.5% ELS and 0.2% *PP* extract. 2CNT = control diet of second cycle; 2ELS5% = 2CNT+ 5% ELS; 2LPP5% = 2CNT + 5% ELS and 0.2% *PP* extract.

**Table 3:** Percentage of fatty acids on total FAME of the experimental diets of the different groups in the two cycles.

Ingredients (%)	First Cycle			Second Cycle		
	1CNT	1ELS5%	1LPP3.5%	2CNT	2ELS5%	2LPP5%
14:0	0.33	0.29	0.32	0.40	0.31	0.28
16:0	16.75	15.74	16.11	16.38	16.08	15.76
16:1 n-7	0.19	0.23	0.21	0.12	0.09	0.07
17:0	0.12	0.11	0.09	0.11	0.10	0.11
18:0	6.80	6.94	7.18	6.86	6.98	7.01
18:1 n-9	19.41	18.63	18.60	26.37	23.88	23.82
18:1 n-7	1.25	1.01	1.20	1.90	1.85	1.79
18:2 n-6, LA _1_	47.25	33.32	33.54	41.12	25.89	26.60
20:0	0.32	0.29	0.26	0.28	0.28	0.22
18:3 n-6, γ-ALA _2_	0.23	0.30	0.25	0.20	0.21	0.22
18:3 n-3, α-ALA	5.35	21.71	20.42	3.90	22.90	22.44
22:1 n-11	0.17	0.17	0.17	0.19	0.22	0.23
20:4 n-6, AA _3_	0.06	0.05	0.19	0.12	0.12	0.16
20:5 n-3, EPA _4_	-	-	0.09	-	-	0.09
22:5 n-3 DPA _5_	-	-	0.06	-	-	0.05
SFA _6_	25.26	24.21	24.85	25.00	24.46	24.15
MUFA _7_	21.79	20.36	20.54	28.97	26.35	26.25
PUFA n-6 _8_	47.60	33.72	34.04	42.14	26.29	27.02
PUFA n3 _9_	5.35	21.71	20.58	3.90	22.90	22.58

^1^ LA = linoleic acid; ^2^ ALA = linolenic acid; ^3^ AA= arachidonic acid, ^4^ EPA = eicosapentaenoic acid; ^5^ DPA = docosapentaenoic acid; ^6^ SFA = saturated fatty acids ∑14:0, 16:0, 17:0, 18:0, 20:0; ^7^ MUFA = monounsaturated fatty acids ∑ 16:1, 18:1n-9, 18:1 n-7, 22:1 n-11; ^8^ PUFA n-6 = polyunsaturated fatty acid omega 6 ∑ 18:2 n-6, 18:3 n-6, 20:4 n-6; ^9^ PUFA n-3 = polyunsaturated fatty acid omega 3 ∑ 18:3 n-3, 20:5 n-3, 22:5 n-3; 1CNT = control diet of first cycle; 1ELS5% = 1CNT+ 5% ELS; 1LPP3.5% = 1CNT+ 3.5% ELS and 0.2% *PP* extract. 2CNT = control diet of second cycle; 2ELS5% = 2CNT+ 5% ELS; 2LPP5% = 2CNT + 5% ELS and 0.2% *PP* extract.

**Table 4:** Live weight and carcass characteristics of rabbits fed on different diets.

		First Cycle (C1)			Second Cycle (C2)						
	1CNT	1ELS5%	1LPP3.5%	*p* Value ^1^	2CNT	2ELS5%	2LPP5%	*p* Value ^2^	RMSE	*p* Value Cycles	*p* Value Groups
LW (g)	2477	2398	2631	ns	2541	2467	2511	ns	233.1	0.137	0.428
CCW (g)	1390.1	1345.3	1369.8	ns	1441.0	1399.9	1436.4	ns	131.5	0.086	0.428
LvW (g)	80.74	76.27	79.26	ns	79.64	81.28	80.69	ns	11.94	0.467	0.877
SFaW (g)	7.68	7.55	8.82	ns	7.69	7.76	7.98	ns	2.67	0.709	0.454
PFaW (g)	9.61	9.31	9.40	ns	10.15	10.07	9.96	ns	2.93	0.304	0.957
FLP (%)	25.22	25.77	25.53	ns	25.04	24.55	24.48	ns	1.25	0.033	0.575
TP (%)	37.49	37.11	37.41	ns	37.23	36.92	37.27	ns	1.54	0.209	0.232
HPP (%)	37.29	37.12	37.06	ns	37.74 ^b^	38.53 ^a^	38.25 ^a^	0.063	1.21	0.008	0.071
HLBP (%)	18.98	19.39	19.45	ns	18.55 ^b^	19.56 ^a^	19.31 ^a^	0.002	0.681	0.603	<0.001
HLFP (%)	1.55	1.46	1.42	ns	1.61	1.51	1.58	ns	0.135	0.042	0.288
HLMP (%)	79.48	79.15	79.14	ns	79.84 ^a^	79.03 ^b^	79.11 ^b^	0.015	0.713	0.321	0.018
M/B	4.19	4.08	4.07	ns	4.30 ^a^	4.04 ^b^	4.10 ^ab^	0.005	0.184	0.378	0.005

^1^ *p* = value between groups of first cycle; ^2^ = *p* value between groups of second cycle; RMSE = root mean square error; a, b = different letters in the same row mean significant difference for *p* < 0.05; ns = non-significant. 1CNT = control diet of C1; 1ELS5% = 1CNT+ 5% ELS; 1LPP3.5% = 1CNT+ 3.5% ELS and 0.2% *PP* extract. 2CNT = control diet of C2; 2ELS5% = 2CNT+ 5% ELS; 2LPP5% = 2CNT with 5% ELS and 0.2% *PP* extract. LW = live weight; CCW = chilled carcass weight; LvW = liver weight; SFaW = scapular fat weight; PFaW= dissectible perirenal and abdominal fat weight; FLP = fore legs; TP = thoracic cage; HPP = hind part; HLBP = hind part separate bone; HLMP = hind part separate meat; HLFP= hind part separate fat and other tissues; M/B = meat-to-bone ratio of the hind leg (HLMP/HLBP).

**Table 5:** Physical characteristics of *Longissimus thoracis et lumborum* of rabbits fed different diets.

		First Cycle (C1)			Second Cycle (C2)						
	1CNT	1ELS5%	1LPP3.5%	*p* Value ^1^	2CNT	2ELS5%	2LPP5%	*p* Value ^2^	RMSE	*p* Value Cycles	*p* Value Groups
pH	5.73	5.75	5.72	Ns	5.83	5.84	5.77	ns	0.123	0.002	0.208
L*	48.32	46.91	47.20	Ns	50.94	51.05	52.39	ns	3.08	<0.001	0.139
a*	−2.45	−2.60	−2.49	Ns	−1.88	−2.17	−2.44	ns	0.728	0.081	0.225
b*	3.28	3.04	3.81	Ns	3.60	3.90	3.21	ns	1.344	0.485	0.979
WHC (%)	13.02	13.13	13.69	Ns	13.98	14.16	14.45	ns	1.34	0.002	0.218
Cooking loss (%)	28.06	27.02	27.38	Ns	26.53	26.34	26.62	ns	1.93	0.014	0.448
WBS (N) raw	12.47	11.78	14.47	Ns	12.93	13.30	13.13	ns	2.75	0.701	0.133
WBS (N) Cooked	21.69	26.56	24.47	Ns	21.99	22.09	20.59	ns	5.47	0.018	0.183

^1^ *p* = value between groups of the first cycle; ^2^ = *p* value between groups of second cycle; RMSE = root mean square error; 1CNT = control diet of C1; 1ELS5% = 1CNT+ 5% ELS; 1LPP3.5% = 1CNT+ 3.5% ELS and 0.2% *PP* extract. 2CNT = control diet of C2; 2ELS5% = 2CNT+ 5% ELS; 2LPP5% = 2CNT with 5% ELS and 0.2% *PP* extract. WHC = water holding capacity, L* = lightness; a* = a index, redness; b* = b index, yellowness; WBS = shear force with Warner–Bratzler dispositive.

**Table 6:** Chemical composition of thigh meat of rabbits fed different diets.

		First Cycle (C1)			Second Cycle (C2)						
	1CNT	1ELS5%	1LPP3.5%	*p* Value ^1^	2CNT	2ELS5%	2LPP5%	*p* Value ^2^	RMSE	*p* Value Cycles	*p* Value Groups
Moisture (%)	75.56	75.63	75.51	ns	75.04	75.08	74.83	ns	0.785	0.005	0.653
ash (%)	1.26	1.21	1.23	ns	1.30	1.28	1.28	ns	0.106	0.019	0.474
Fat (%)	2.50	2.59	2.40	ns	2.80	2.92	2.87	ns	0.443	<0.001	0.504
Protein (%)	20.68	20.57	20.85	ns	20.86	20.72	21.01	ns	0.718	0.255	0.284
Vitamin E (mg/100 d)	2.38 ^b^	2.56 ^b^	3.09 ^a^	<0.001	2.26 ^c^	2.61 ^b^	3.31 ^a^	<0.001	0.426	0.547	<0.001
Q10 (mg/100 g)	19.24 ^b^	20.33 ^a^	20.09 ^ab^	0.046	19.61 ^b^	20.34 ^a^	20.54 ^a^	0.031	1.251	0.289	0.006
Cholesterol (mg/100 g)	50.87	50.36	50.24	ns	50.65 ^ab^	50.98 ^a^	48.83 ^b^	0.054	2.76	0.551	0.109

^1^ *p* value between groups of first cycle; ^2^ *p* value between groups of second cycle; RMSE = root mean square error; a, b = different letters in the same raw means significant difference for *p* < 0.05; ns = non-significant value. 1CNT = control diet of C1; 1ELS5% = 1CNT+ 5% ELS; 1LPP3.5% = 1CNT+ 3.5% ELS and 0.2% *PP* extract. 2CNT = control diet of C2; 2ELS5% = 2CNT+ 5% ELS; 2LPP5% = 2CNT with 5% ELS and 0.2% *PP* extract.

**Table 7:** Principal fatty acid classes expressed as percentage of total FAMEs on thigh meat of rabbits fed different diets.

		First Cycle (C1)			Second Cycle (C2)						
	1CNT	1ELS5%	1LPP3.5%	*p* Value ^1^	2CNT	2ELS5%	2LPP5%	*p* Value ^2^	RMSE	*p* Value Cycles	*p* Value Groups
SFA	36.30 ^a^	34.35 ^b^	35.09 ^ab^	<0.001	35.83 ^a^	34.52 ^b^	33.34 ^c^	<0.001	1.223	0.007	<0.001
MUFA	23.77	24.88	24.07	ns	29.88 ^a^	28.11 ^b^	27.08 ^c^	<0.001	1.576	<0.001	0.006
PUFA n-6	35.63 ^a^	29.09 ^b^	29.83 ^b^	<0.001	30.18 ^a^	24.58 ^c^	26.38 ^b^	<0.001	1.601	<0.001	<0.001
PUFA n-3	4.06 ^b^	11.46 ^a^	10.78 ^a^	<0.001	3.93 ^b^	12.57 ^a^	12.86 ^a^	<0.001	0.954	<0.001	<0.001
PUFA	39.88	40.72	40.79	ns	34.20 ^c^	37.26 ^b^	39.44 ^a^	<0.001	1.545	<0.001	<0.001
n-6/n-3	9.05 ^a^	2.57 ^b^	2.80 ^b^	<0.001	8.10 ^a^	1.98 ^b^	2.06 ^b^	<0.001	1.243	0.003	<0.001

^1^ *p* = value between groups of the first cycle; ^2^ = *p* value between groups of second cycle; RMSE = root mean square error; a, b, c = different letter in the same row means significant difference for *p* < 0.05; ns = non-significant value. SFA = saturated fatty acid (10:00, 12:00, 13:00, 14:00, 15:0iso, 14:01, 15:0anteiso, 15:00, 16:0iso, 16:00, 17:0iso, 17:0anteiso, 17:00, 18:0iso, 18:00, 20:00, 21:00, 22:00 23:00, 24:00); MUFA = monounsaturated fatty acid (14:1, 15:1, 16:1n-9, 16:1n-7, 17:1 n-7, 18:1trans-9, 18:1trans-11, 18:1n-9, 18:1n-7, 20:1n-9, 20:1n-7, 22:1n-11, 24:1n-15); PUFA n-6 = polyunsaturated fatty acids omega-6 (18:2n6, 18:2n6 trans isomer, conjugate linoleic acids (CLAs), 18:3 n-6, 20:2 n-6, 20:3 n-6, 20:4 n-6, 22:2 n-6, 22:4 n-6); PUFA n-3 = polyunsaturated fatty acids omega 3 (18:3 n-3, 20:3 n-3, 20:5 n-3, 22:5 n-3, 22:6 n-3); 1CNT = control diet of C1; 1ELS5% = 1CNT+ 5% ELS; 1LPP3.5% = 1CNT+ 3.5% ELS and 0.2% *PP* extract. 2CNT = control diet of C2; 2ELS5% = 2CNT+ 5% ELS; 2LPP5% = 2ELS5% with 5% ELS and 0.2% *PP* extract.

**Table 8:** Macro- and micromineral elements on rabbit thigh meat in two cycles and different diets.

		First Cycle (C1)			Second Cycle (C2)						
	1CNT	1ELS5%	1LPP3.5%	*p* Value ^1^	2CNT	2ELS5%	2LPP5%	*p* Value ^2^	RMSE	*p* Value Cycles	*p* Value Groups
P (mg/100 g)	238.83	240.65	240.72	ns	238.92 ^b^	242.98 ^ab^	246.92 ^a^	0.014	7.555	0.116	0.035
K (mg/100 g)	377.79	380.37	379.97	ns	380.08	383.65	382.12	ns	8.36	0.135	0.328
NA (mg/100 g)	56.69	57.40	57.23	ns	56.91	56.35	56.05	ns	2.196	0.138	0.909
Mg (mg/100 g)	24.94	25.20	25.15	ns	25.03	25.51	25.39	ns	1.07	0.336	0.311
Mn (mg/kg)	0.34	0.33	0.33	ns	0.35	0.33	0.33	ns	0.029	0.094	0.233
Fe (mg/kg)	4.32	4.17	4.29	ns	4.36	4.23	4.42	ns	0.291	0.193	0.08
Zn (mg/kg)	11.27 ^b^	11.90 ^ab^	12.26 ^a^	0.022	11.42 ^b^	11.87 ^ab^	12.52 ^a^	0.012	1.015	0.538	<0.001
Cu (mg/kg)	0.83	0.91	0.89	ns	0.97	0.90	0.94	ns	0.096	0.004	0.843

^1^ *p* = value between groups of first cycle; ^2^ P = value between groups of second cycle. RMSE = root mean square error; ^ab^ = different letter in the same row means significant difference for *p* < 0.05; ns = non-significant value. P mg/100 g = Phosphorus; K mg/100 g = Potassium; Na mg/100 g = Sodium; Mg mg/100 g = Magnesium; Mn mg/kg = Manganese; Fe mg/kg = Iron; Zn mg/kg = Zinc; Cu mg/kg = Copper; 1CNT = control diet of C1; 1ELS5% = 1CNT+ 5% ELS; 1LPP3.5% = 1CNT+ 3.5% ELS and 0.2% *PP* extract. 2CNT = control diet of C2; 2ELS5% = 2CNT+ 5% ELS; 2LPP5% = 2ELS5% with 5% ELS and 0.2% *PP* extract.

## Data Availability

The original contributions presented in the study are included in the article.
